# Unilateral biportal endoscopic vs. open surgery in the treatment of young obese patients’ lumbar degenerative diseases: a retrospective study

**DOI:** 10.3389/fsurg.2024.1467768

**Published:** 2024-10-29

**Authors:** Tao Ma, Junyang Li, Yongcun Geng, Dengming Yan, Ming Jiang, Xiaoshuang Tu, Senlin Chen, Jingwei Wu, Luming Nong

**Affiliations:** ^1^ Department of Orthopedics, The Affiliated Changzhou No.2 People's Hospital of Nanjing Medical University, Changzhou Medical Center, Nanjing Medical University, Changzhou, China; ^2^ Department of Orthopedics, Nanjing Medical University, Jiangsu, China; ^3^ Department of Orthopedics, Dalian Medical University, Liaoning, China

**Keywords:** minimally invasive surgery, lumbar degenerative disease, UBE, lumbar surgery, spine

## Abstract

**Background:**

Obesity accelerates the development of lumbar disease and increase the risk during surgery. Unilateral biportal endoscopic discectomy (UBE) is a newly developed minimally invasive technique, which refers to the spinal surgery under unilateral double-channel endoscopic surgery. Therefore, the purpose of this study is whether UBE decompression alone can bring good clinical results to young obese patients with lumbar degenerative diseases.

**Methods:**

The patients with lumbar diseases who underwent UBE and open surgery (open discectomy) in our hospital from February 2020 to February 2022 were selected as young (age ≤ 44 years old) and obesity (BMI ≥ 30 kg/m^2^). The patients were evaluated with VAS, ODI, JOA and modified Macnab score before operation, 1 month, 6 months and 12 months after operation. Nerve root function sensation, muscle strength and tendon reflex were evaluated. The operation time, estimated blood loss, postoperative hospital stay, incidence of postoperative complications and reoperation rate were recorded. MRI quantitative lumbar multifidus muscle (LMM) comparison was performed 12 months after operation.

**Results:**

77 patients were included, and the scores of VAS, ODI and JOA were similar in the two groups during the last follow-up. There were no difference in nerve root function sensation, muscle strength or tendon reflex. However, one month after operation, the VAS back score and ODI improvement in the UBE group were significantly better than those in the open group, which were 2.44 ± 0.97, 33.10 ± 6.78 and 2.93 ± 0.79 and 36.13 ± 5.84, respectively, with a statistically significant difference (*p* = 0.020 and 0.038). According to the modified Macnab criteria, UBE group, the excellent and good rate was 97.2%. The excellent and good rate of open group was 97.6%. The estimated blood loss and postoperative hospital stay in UBE group (36.81 ± 17.81, 3.92 ± 1.32) were significantly better than those in open group (104.88 ± 31.41, 6.41 ± 1.94), with a statistically significant difference (*p* = 0.010). There was no significant difference in operation time between the two groups (*p* = 0.070). The number of complications in UBE group was 2 (5.6%) and open group was 4 (9.8%). The fat infiltration rate of 19.3%+11.0% in UBE group was significantly lower than that of 27.0%±13.9% in open group (*p* = 0.010).

**Conclusion:**

UBE has the advantage of early recovery in the treatment of lumbar degenerative diseases in young obese patients, and reduces the damage to LMM, so it has a good clinical effect.

## Introduction

Low back pain (LBP) is a common symptom of lumbar spine diseases such as lumbar spondylolisthesis, lumbar disc herniation, and lumbar spondylolysis (1). Both conservative and surgical treatments cannot fundamentally reverse the degeneration of the lumbar spine, but can only alleviate pain and slow down the progression of the disease (2). Low back pain in the elderly population is often related to degenerative diseases of the lumbar spine, while degeneration in young people is often related to lifestyle factors such as prolonged incorrect posture (3). Overweight and obesity are not only associated with diabetes and cardiovascular diseases, but have also been shown in multiple studies to be one of the important risk factors for causing bone and joint diseases (4, 5). Overweight patients who stand or sit for a long time increase the load on the lumbar spine, accelerating its degeneration rate (6). One study found that the bone marrow density of obese patients (BMI ≥ 30 kg/m^2^) under magnetic resonance imaging was reduced (7). Moreover, some studies have found that as weight is gained, the proportion of macrophages infiltrating the adipose tissue increases. Then, as a result of biochemical interaction between the adipocytes and macrophages, cascades of production of proinflammatory cytokines, known as adipokines, 6 are activated. More than 50 adipokines are currently known; among the most studied are leptin, adiponectin, resistin and the so-called RBP-4. These substances cause numerous metabolic, inflammatory and immune system effects that produce chronic inflammatory changes in the disc (8).

Obesity cannot be a contraindication for surgery (9), but some authors believe that obesity is an independent factor that can prolong surgical time, increase blood loss, increase the risk of infection, and increase the probability of postoperative complication (10, 11). Therefore, surgeons prefer to give the obesity group conservative management, instead of operational management. When patients have severe pain symptoms after failing to respond to conservative treatment, they often choose surgical treatment. Traditional open surgery causes larger wounds and more muscle damage, which is not conducive to patient recovery. With the continuous development of spinal surgery methods, minimally invasive endoscopic techniques have gradually matured and are widely used in clinical practice (12). Minimally invasive techniques not only bring psychological comfort to patients, but also have good surgical effects with less bleeding, shorter duration of pain, and faster postoperative recovery (13). Unilateral biportal endoscopic (UBE) technology has been proven to bring good postoperative recovery for degenerative diseases of the lumbar spine (14, 15). Some authors believe that the effect of simple decompression surgery in obese patients is worse than that in normal people, and obesity should be considered an important factor for fusion surgery (16). However, for young obese patients, degeneration in adjacent segments are prone to occur during long-term survival after fusion surgery (17). We believe that compared with other minimally invasive techniques, UBE can achieve more extensive and thorough decompression. Therefore, the purpose of this article is to study whether UBE technique can bring good recovery effects to young obese patients with degenerative diseases of the lumbar spine, protect the LMM, and reduce complications and recurrence rates.

## Method

**Figure 1:**
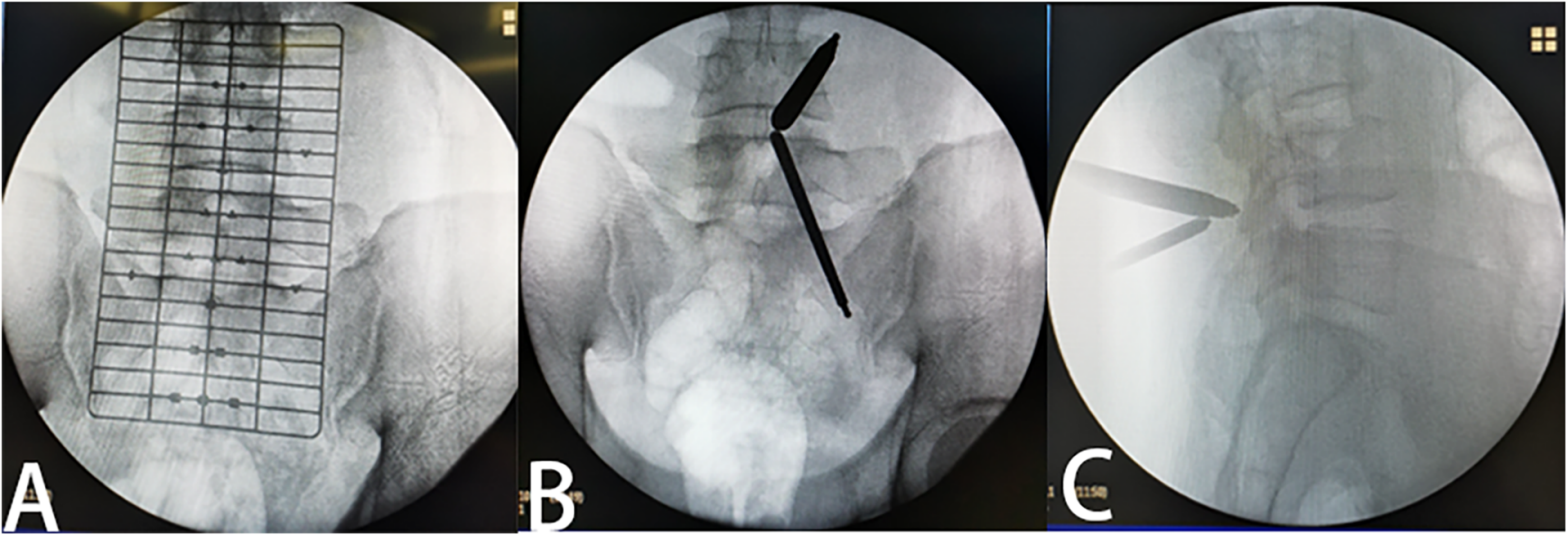
Intraoperative localization **(A)** preoperative positioning perspective. **(B)** Forward perspective after the channel is established. **(C)** Lateral perspective after the channel is established.

This study has been approved by the local ethics commit [(2023)KY027-01]. The patients who were treated with UBE technique and traditional open surgery in our hospital from February 2020 to February 2022 were analyzed. Inclusion criteria: 1. The young patients with no response to conservative treatment (age ≤ 44 years old) (18). 2. BMI ≥ 30 kg/m^2^. 3. Combined with Magnetic Resonance Imaging (MRI) and Computed Tomography (CT), lumbar spinal canal stenosis or lumbar disc herniation was diagnosed. 4. Patients who have undergone UBE or open discectomy or decompression for the first time. Exclusion criteria: 1. With segmental instability or lumbar spondylolisthesis. 2. Multiple surgeries. VAS, ODI, JOA and modified Macnab score were used to evaluate the clinical efficacy of the patients before operation and 1, 6, 12 months after operation. The operation time, estimated estimated blood loss, postoperative hospital stay, postoperative complications and recurrence rate within 1 year were recorded. One year after operation, LMM area, LMM fat infiltration area and LMM fat infiltration rate were measured by MRI quantitative analysis to evaluate the changes of LMM in the two groups. Image J software was used to process MRI images. Borders of the LMM were delineated manually as the software allows measuring in a random shaped region of interest. According to different pixel thresholds, the area of fat infiltration can be automatically screened, and finally the fat infiltration rate can be calculated (Figure 1). All measurement results were averaged by three authors.

## Statistics

Data were statistically described in terms of mean ± standard deviation (SD), or frequencies (number of cases) and percentages when appropriate. Categorical variables were analyzed with a Chi-squared test. Independent sample *t*-test was used between groups, *p* < 0.05 was statistically significant. We used SPSS 22.0 for statistical analysis.

## Surgical procedure

### UBE

**Figure 2:**
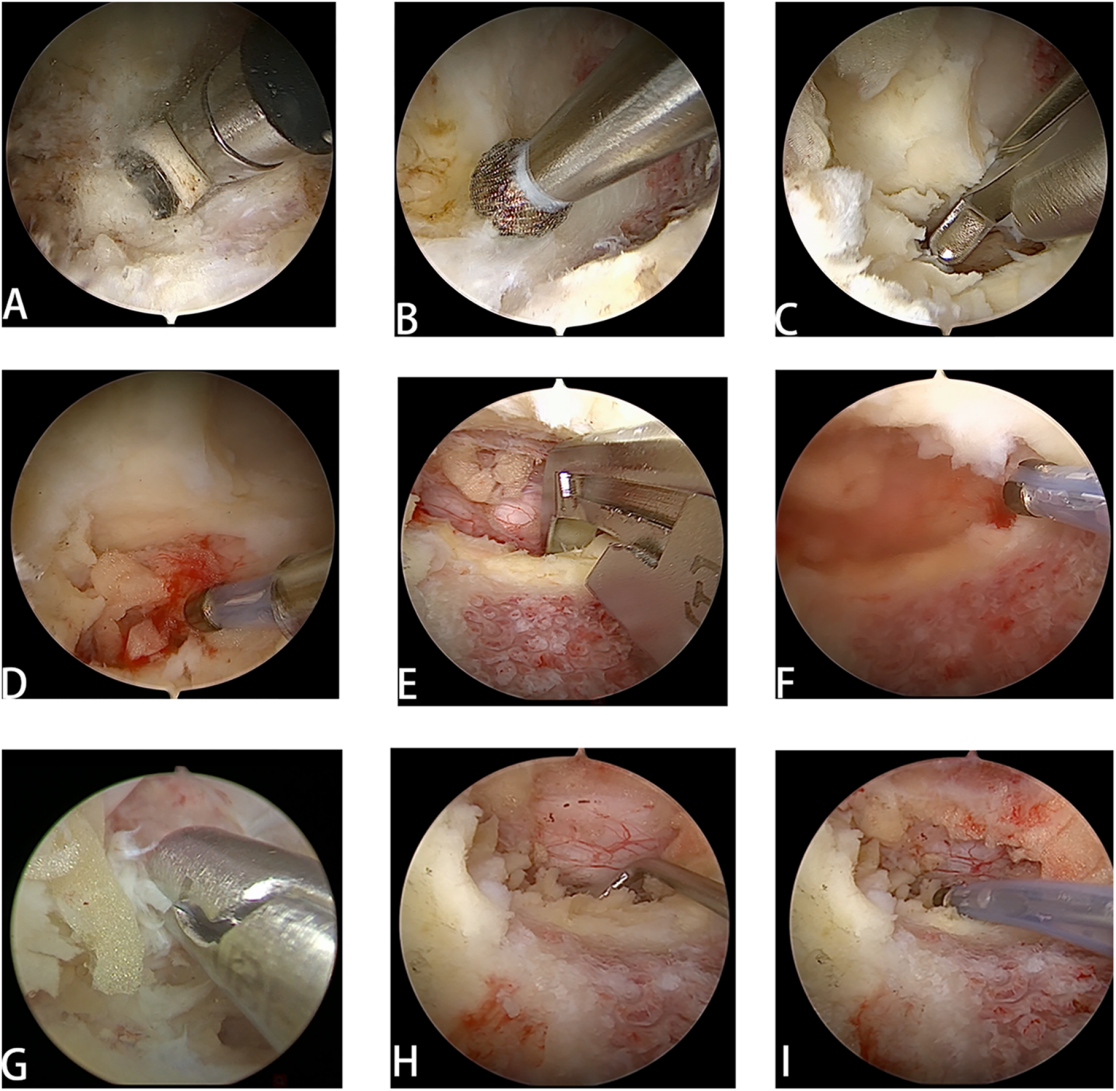
UBE surgical procedure. **(A)** The channel was established and the soft tissue was removed. **(B)** The drill reaches the surface of the lamina and readies to grind out the lamina. **(C)** The ligamentum flavum was resected for decompression. **(D)** Use electric knife to stop bleeding promptly and prevent excessive bleeding. **(E)** Endoscopic laminectomy was performed according to the range of decompression. **(F)** High frequency electrotome was used to stop blood vessels on the surface of the dural sac. **(G)** The intervertebral disc was removed. **(H)** Use a nerve retractor to release the nerve roots. **(I)** Retrace the nerve.

In the prone position after general anesthesia, the interlaminar space was opened by cushion support. The bed was ajusted to the right height for the surgeon. The positioning film was used to determine the responsibility gap under the perspective of the C-arm (Figure 1). After routine disinfection of the sheet, a waterproof curtain was set up to prevent soaking the operation sheet. We chose the responsibility space as the center and made about two longitudinal incisions of 1 cm at the medial edge of the superior and inferior pedicles. The dilator was placed from the incision to the lamina, and the location of the passage will be determined by fluoroscopy under the C-arm again, especially in the case of hypertrophic back fat, to prevent us from losing the correct anatomical relationship. Continuous infusion of isotonic saline is applied to maintain the surgical visual field. After removing the soft tissue that hinders the visual field, the anatomical relationship between the lamina and the ligamentum flavum was fully exposed. Kerrison punch and a grinding drill were used to remove the lamina until the root of the ligamentum flavum. Then we explored the ligamentum flavum to ensure that there is no adhesion to the dural sac, used Kerrison punch to remove the ligamentum flavum and fully exposed the dural sac. In this process, a small electric knife was used to stop bleeding or pre-stop bleeding in time to reduce the amount of bleeding during the operation. When the dural sac was exposed, the nerve was explored to determine the location of nucleus pulposus compression. Nerve retractor was used to pull the dural sac to expose the protruding nucleus pulposus, and nucleus pulposus clamp was used to remove the nucleus pulposus. At last, we explored the dural sac or nerve root again to ensure complete release (Figure 2).

### Open discectomy

After anesthesia, the patient was placed prone on the operating table. The next steps were as follows: 1. Routine disinfection with sheets placement. 2. Midline longitudinal incision centered on the responsibility space. 3. Sequential incision of the skin, subcutaneous tissue, and deep fascia. 4. Full exposure of the paravertebral plate and responsibility space. 5. Removal of the responsibility plate and the ligamentum flavum. 6. Exploration of the dural sac and nerve root after entering the spinal canal. 7. Removal and dissociation of the nucleus pulposus as needed to complete the neurolysis.

## Results

A total of 77 patients met the criteria, 36 patients in the UBE group, the age was 37.92 ± 6.32 years old, and the BMI was 34.88 ± 3.04. 41 Patients in open group, age 40.61 ± 6.90, BMI was 35.12 ± 2.99. there was no significant difference in baseline data between the two groups (Table 1).

**Table 1:** Patient characteristics.

Variables	Open group	UBE group	
	(*n* = 41)	(*n* = 36)	*P* value
Sex (Male/Female)	18/23	18/18	0.637
Age (years)	40.61 ± 6.90	37.92 ± 6.32	0.079
BMI (m/kg^2^)	35.12 ± 2.99	34.88 ± 3.04	0.735
Number of segments			0.300
Single segment	38	33	
Double segment	3	3	
Diagnosis			0.163
Lumbar intervertebral disc	30	31	
Lumbar spinal stenosis	11	5	
Neurologic symptoms			
Nerve root function sensation			0.787
Normal	32	29	
Abnormal	9	7	
Muscle strength			0.640
Normal	30	28	
Abnormal	11	8	
Tendon reflex			0.132
Normal	31	32	
Abnormal	10	4	

The operation time in UBE group was 70.65 ± 22 and 61.95 ± 19.07 min in UBE group. There was no significant difference in operation time between the two groups (*p* = 0.070). However, the estimated blood loss in UBE group was significantly less than that in open group (*p* < 0.001). The postoperative hospital stay in UBE group was 3.92 ± 1.32 d, open group was 6.41 ± 1.94 d, there was significant difference (*p* < 0.001) (Table 2).

**Table 2:** Comparison of operative parameters between the two groups.

Group	Open group	UBE group	
	(*n* = 41)	(*n* = 36)	*P* value
Operation time (min)	61.95 ± 19.07	70.65 ± 22.00	0.070
Estimated blood loss (ml)	104.88 ± 31.41	36.81 ± 17.81	0.000*
Postoperative hospital Stay (day)	6.41 ± 1.94	3.92 ± 1.32	0.000*

* Significant difference between two groups.

The VAS, ODI and JOA scores of the two groups were significantly improved during the follow-up, and there was no significant difference between the two groups at the last follow-up (Table 3). At one month after operation, there were significant differences in VAS back score and ODI improvement between UBE group and open group (*p* values are 0.020 and 0.038, respectively). There was no significant difference in other follow-up periods.

**Table 3:** Comparison of clinical outcomes between the two groups.

	Open group	UBE group	
	(*n* = 41)	(*n* = 36)	*P* value
VAS back scores			
Preoperative	5.12 ± 1.45	5.67 ± 1.26	0.085
1 month after operation	2.93 ± 0.79	2.44 ± 0.97	0.020*
6 months after operation	1.61 ± 0.77	1.58 ± 0.73	0.878
12 months after operation	0.71 ± 0.56	0.86 ± 0.59	0.245
VAS leg scores			
Preoperative	5.76 ± 1.02	5.58 ± 1.00	0.456
1 month after operation	2.49 ± 1.00	2.86 ± 0.76	0.073
6 months after operation	1.59 ± 0.89	1.86 ± 0.76	0.152
12 months after operation	0.73 ± 0.87	1.00 ± 0.48	0.092
ODI scores			
Preoperative	61.96 ± 9.22	63.44 ± 9.89	0.499
1 month after operation	36.13 ± 5.84	33.10 ± 6.78	0.038*
6 months after operation	17.21 ± 4.71	18.86 ± 5.24	0.149
12 months after operation	5.60 ± 4.24	5.09 ± 2.89	0.550
JOA scores			
Preoperative	9.95 ± 2.11	9.86 ± 1.84	0.843
1 month after operation	17.85 ± 2.13	18.47 ± 1.70	0.167
6 months after operation	20.27 ± 1.57	20.75 ± 1.54	0.178
12 months after operation	22.12 ± 2.15	23.06 ± 2.11	0.059

* Significant difference between two groups.

**Figure 3:**
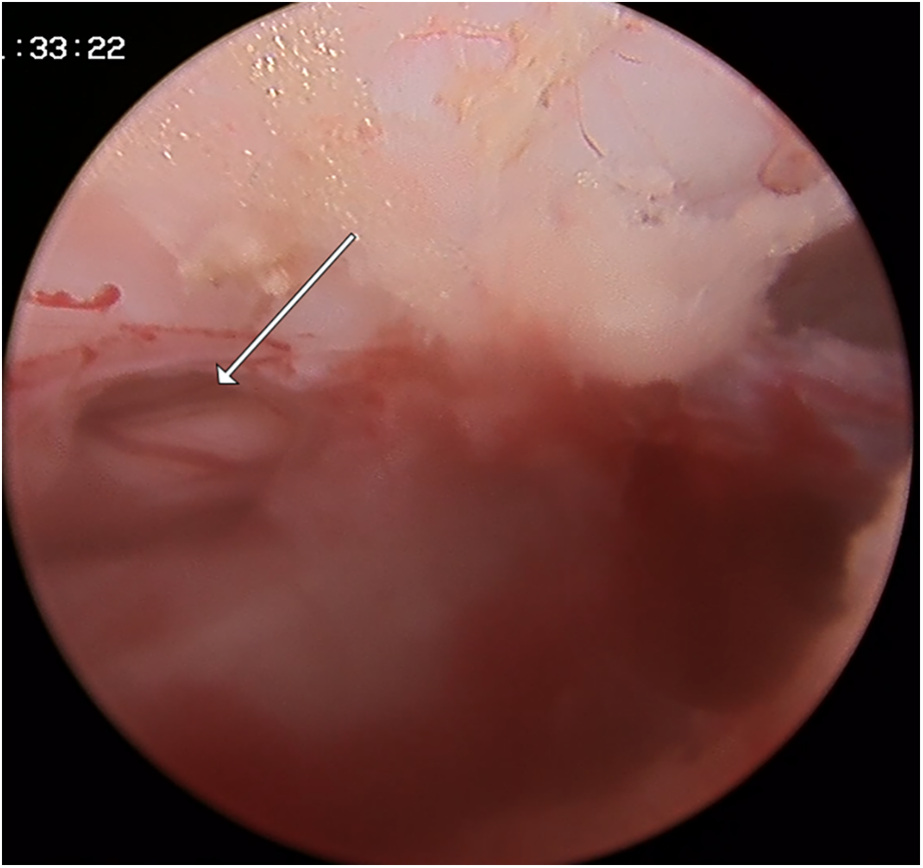
A 35-year-old man developed a ruptured dural sac in UBE group. Because of the small size of the breach, only the drainage tube was placed and no suture was performed.

At the last follow-up, according to the modified Macnab criteria, UBE group 30 cases were excellent, 5 cases were good, 1 case was poor, and the excellent and good rate was 97.2%. The open group excellent and good rate of open group was 97.6% (32 cases were excellent, 8 cases were good and 1 case was poor). There were 2 (5.6%) complications in UBE group and 4 (9.6%) complications in open group (Table 4; Figure 3). None of the patients in the two groups received reoperation within 12 months after operation. According to the patient's symptoms and radiological data, 3 patients (7.3%) in the open disectomy group and 1 person (2.8%) in the UBE group recurred at 1 year after operation. Quantitative analysis of LMM by MRI showed that there was no significant difference in LMM area between the two groups (*p* = 0.731), but the LMM fat infiltrating area in the UBE group was significantly less (*p* = 0.044). The LMM fat infiltration rate of patients in the UBE group was 19.3% + 11.0% and had no significant difference before operation (*p* = 0.112), while that of patients in the open group was 27.0% ± 13.9% and the difference before operation was statistically significant (*p* < 0.001), and the difference of fat infiltration rate between the two groups was statistically significant (*p* = 0.010) (Table 5). The benefits of UBE are show in young obese patients, especially early recovery and protection of the LMM.

**Table 4:** Comparison of complications the two groups.

Complications (%)	OPEN (*n* = 41)	UBE (*n* = 36)
Dural tear	1 (2.4)	1 (2.8)
Surgical site infection	1 (2.4)	0 (0.0)
Hematoma	1 (2.4)	0 (0.0)
Incomplete decompression	1 (2.4)	1 (2.8)
Other	0 (0.0)	0 (0.0)

**Table 5:** Comparison of LMM changes between the two groups.

	Open group	UBE group	*P* value
Pre-op LMM area	6,783.15 ± 1,841.18	6,682.94 ± 1,789.95	0.810
Pre-op LMM fat infiltrated area	1,338.51 ± 632.36	1,275.89 ± 926.07	0.727
Pre-op Fat infiltration rate	20.70%±10.50%	18.30%±10.40%	0.321
Post-op LMM area	6,714.24 ± 1,844.03	6,573.00 ± 1,727.13	0.731
Post-op LMM fat infiltrated area	1,743.66 ± 861.65	1,318.58 ± 958.87	0.044*
Post-op fat infiltration rate	27.00%±13.90%	19.30%+11.00%	0.010*

* Significant difference between two groups.

## Discussion

In this study, the sample is small although some of the data is statically significant. we confirmed that UBE technique and open surgery have similar clinical results in obese patients, but the incidence of complications in UBE group is lower. Young and obese patients can be discharged from hospital more quickly after UBE decompression surgery, and VAS back and ODI have better performance than open surgery one month after operation. Not only that, UBE caused little damage to the low back muscles, patients get out of bed as soon as possible to exercise the low back muscles, which is conducive to the recovery of the disease, forming a virtuous circle.

More and more studies show that obesity may accelerate lumbar degeneration. A systematic review analysis shows that people with high BMI have twice the risk of LBP (19), and many literatures have confirmed that obesity is an important factor leading to prolonged surgery, increased bleeding, infection and venous thrombosis (20–22), so obese patients are people we should pay more attention to. According to previous studies, obesity affects lumbar degenerative diseases in many ways, but no consensus has been reached on the specific mechanism. Marinko et al. believe that endplate defects are the initial factor of intervertebral disc degeneration (23), because endplate integrity is very important for the maintenance of intervertebral disc environment, the increase of BMI is significantly related to intervertebral disc degeneration, and the slight interruption of endplate structural integrity of intervertebral disc degeneration is enough to cause significant changes in mechanical pressure in the intervertebral disc and the living environment of nucleus pulposus (24). This effect is particularly significant in obese patients when the endplate is destroyed, the environment without blood vessels of the intervertebral disc is also destroyed, and the higher pro-inflammatory factors in the blood of obese patients will accelerate the degeneration of the intervertebral disc. An *in vitro* study by nestorg et al. pointed out that there are significant differences in axial and compression biomechanics between obese patients and non-obese patients, and this difference is more prominent in women (25). The change of abnormal mechanical load can lead to annulus fibrosus tear, which gives more opportunities for nucleus pulposus to protrude outward, which is also an important factor affecting intervertebral disc degeneration in obese patients.

The treatment of lumbar degenerative diseases in obese patients is controversial. MarieT et al. used minimally invasive lumbar foramen lumbar fusion to treat lumbar degenerative diseases in elderly obese patients and achieved good results (26). For young obese patients, premature interbody fusion may be a potential factor for adjacent segmental degeneration in the future. For young obese patients, we do not advocate premature fusion surgery before the emergence of fusion indications such as segmental lumbar instability and lumbar spondylolisthesis, but choose adequate nerve decompression to relieve symptoms.

**Figure 4:**
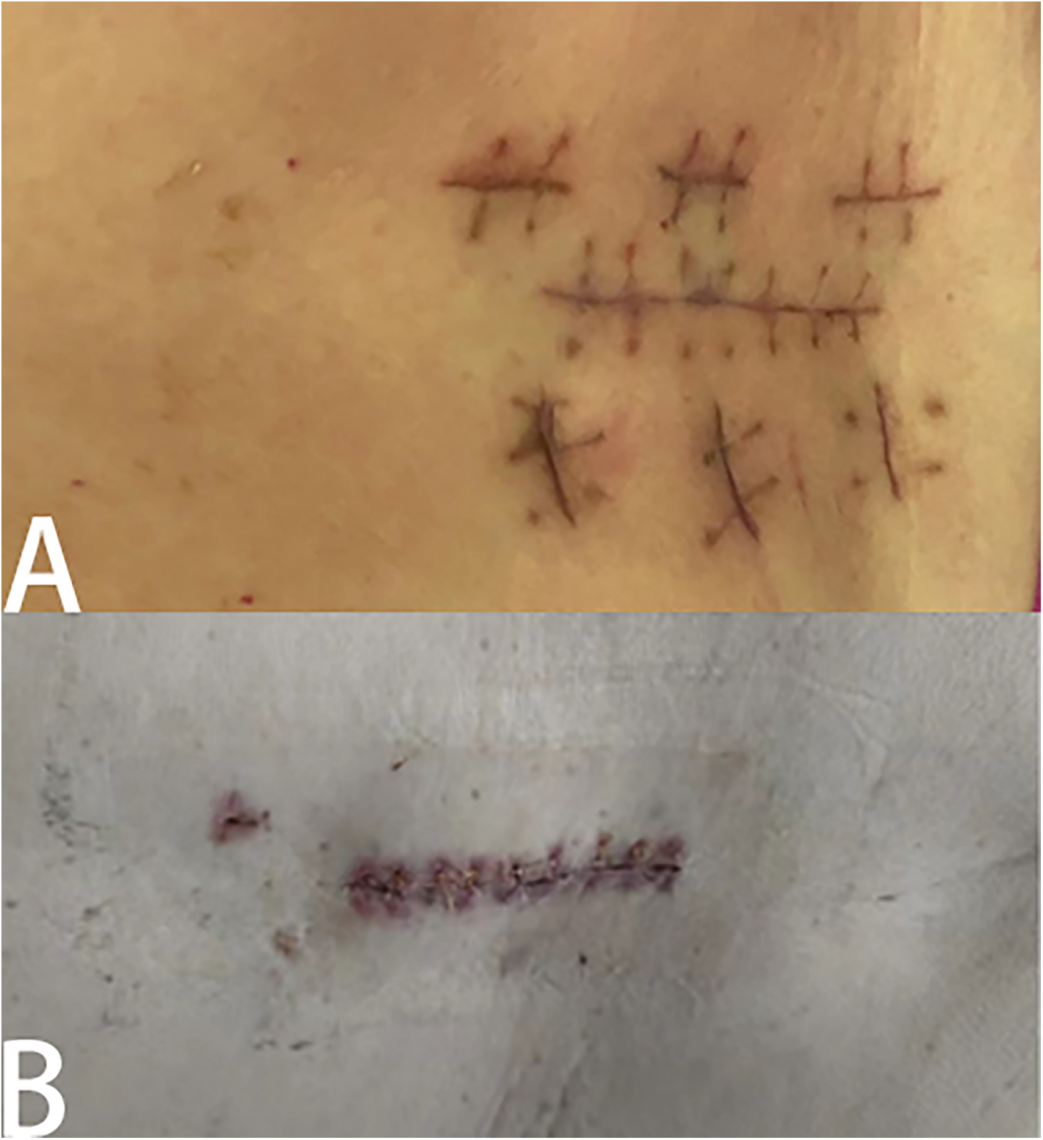
Results of different surgical incisions. Different situations when something unexpected happens during surgery and you have to switch to open surgery. **(A)** When a transverse incision is made, an additional longitudinal incision will be made, rendering the wound ugly. **(B)** The longitudinal incision can be directly connected to form an open incision.

With the development of minimally invasive technology, the concept of minimally invasive has been deeply rooted in the hearts of the people. UBE technique has been widely used in lumbar surgery. It is decompressed by posterior lamina approach. Compared with open surgery, UBE, as a minimally invasive technique, has the characteristics of less trauma, less bleeding and rapid recovery after operation, which enables patients to get out of bed early and prevent lower limb venous thrombosis and bedridden pneumonia. Although some studies have suggested that single-channel endoscopy has less damage to soft tissue and has achieved good clinical results in lumbar degenerative diseases. However, the authors believe that, first of all, dual-channel endoscopy is easier to operate than single-channel endoscopy, and it is easier for doctors with endoscopic basis to master, and UBE is more flexible and has a wider range of activities in the face of patients with hypertrophic low back fat, and can achieve more extensive decompression such as contralateral decompression and lateral recess decompression. During surgery, obese patients are more likely to have complications than the general population, so surgeons are required to operate more carefully, so we choose UBE technique to perform fine operations on nerves under continuous saline infusion to maintain a clear surgical field of vision, so as to minimize iatrogenic injury. In addition, compared with open surgery, UBE reduces unnecessary bone destruction and maintains the stability of lumbar vertebrae, which is beneficial for obese patients to bear greater pressure load when they get out of bed. Different from Heo et al. (27), in order to maximize the impact of hypertrophic back fat on the operation and increase the success rate of the operation, we use our improved longitudinal incision (Figure 4). One is that the longitudinal incision is more consistent with the direction of muscle and fascia than the transverse incision, with less damage to it. Second, in the face of thicker back fat, there is a slight deviation between the fluoroscopic position and the body surface position, when the position of the incision we choose is not satisfactory (for example, upper or lower), the incision can be appropriately extended to avoid the injury caused by the new incision (Figure 4) from the face. In addition, when the operation is accidentally forced to change to open surgery, the two channels can be directly connected to form a new open incision, which can not only avoid ugly surgical wounds but also reduce postoperative wound pain.

Intervertebral disc calcification is a common clinical phenomenon, which is more common in young patients with lumbar disc herniation. Some scholars believe that intervertebral disc calcification is a part of intervertebral disc degeneration, and the degree of intervertebral disc degeneration is similar to the degree of calcification (28), but the specific reason has not been agreed. Benneke et al. believe that endplate morphology also affects the calcification of intervertebral disc, and the degree of endplate degeneration is positively correlated with the degree of intervertebral disc calcification (29). In people with higher BMI, there are higher levels of inflammatory cytokines in blood, which can promote the release of vascular endothelial growth factor, promote neovascularization, promote granulation tissue wrapping, increase osteopontin expression, accelerate calcium and phosphorus deposition, thus promote intervertebral disc calcification. Neovascularization brings more inflammatory factors, which creates a vicious circle and accelerates the degeneration of intervertebral disc. Ectopic calcified intervertebral disc tissue is often attached to the dura mater, resulting in oppressive symptoms. The clinical significance of calcification after disc herniation is not clear, Some scholars believe that it is also a kind of degeneration. For young patients, the demand for daily life activities is greater, and obesity is a risk factor for this degeneration. The UBE technique can make the operation refined, completely visualize and minimize the paravertebral muscle tissue, minimize the damage to the spinal ligament, muscle and bone structure, and help to reduce postoperative pain, promote functional recovery and ensure the safety of the operation (30).

**Figure 5:**
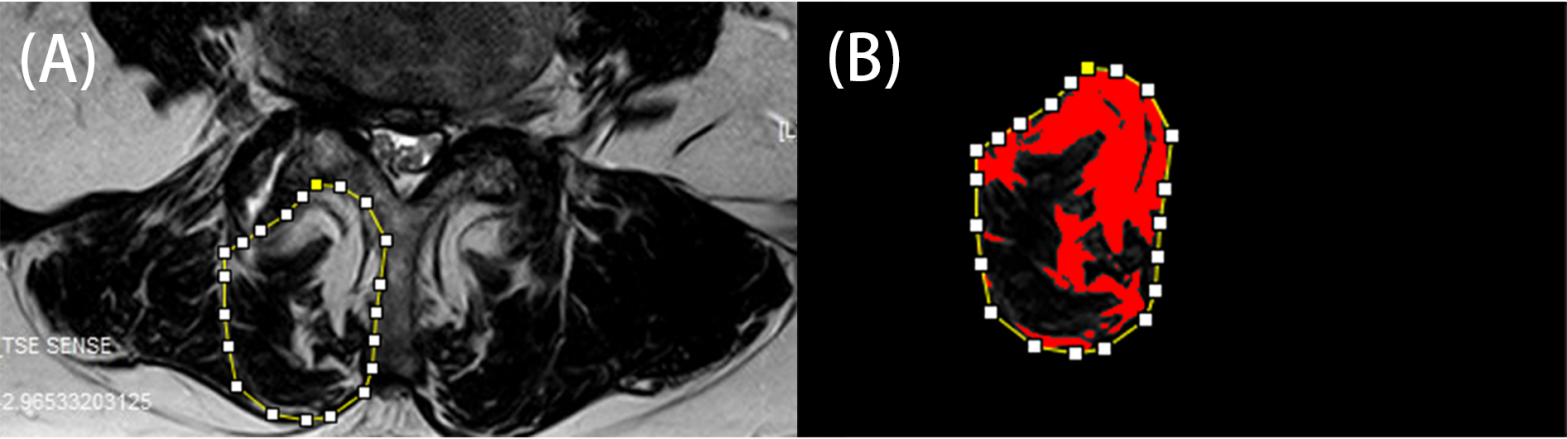
LMM similar in shape and quality. **(A)** Preoperative MRI examination. **(B)** Postoperative MRI examination.

Any injury caused by surgery is likely to be more pronounced in obese people. UBE technique is more invasive than single-channel endoscopy. although there are only two smaller wounds on the surface of the skin, it also causes some damage to fascia and muscles. Lumbar multifidus muscle (LMM) plays an important role in spinal movement and maintaining spinal stability. For spinal movement, LMM injury is more serious than any muscle injury. A number of reports have confirmed that fat infiltration of cleft muscles is associated with low back pain (31–33). One of the studies shows that women have a higher prevalence of LMM fat infiltration. However, in their study, obesity alone did not cause fat infiltration in LMM. However, patients who exercised less and sat for long periods of time had a higher incidence of LMM fat infiltration even without obesity (34). This means that we should encourage patients to get out of bed and exercise their low back muscles as soon as possible. A number of studies have reported LMM damage caused by open surgery (35), such as LMM atrophy and fat infiltration. Therefore, we collected the data of MRI examination more than 3 months after operation in 12 cases in UBE group. The shape and quality of LMM were observed. Through visual evaluation, it was found that the cross-sectional area of LMM did not decrease significantly and there was no obvious fat infiltration (Figure 5). UBE can not only cause less damage to low back muscles, but also make patients with functional exercise in advance, which is helpful to reduce the injury of low back muscles and relieve the symptoms of LBP. Therefore, we think that UBE technique is more suitable for the treatment of lumbar disease in young obese patients. This study has several limitations: First of all, this is a retrospective study with a small number of samples, followed by a short follow-up period. A prospective randomized controlled study with longer follow-up time and larger sample size is needed.

Our study's limitations include strict patient selection, a small number of cases, short-term follow-up, and a lack of a control group. Large-scale studies with more patients, a long-term follow-up, and a comparative study with other surgical techniques are necessary to prove that UBE is more suitable for lumbar disc herniation in young obese patients.

## Conclusion

UBE technique has a wide range of operations, less trauma, a wide field of vision, and reduces the damage to LMM, which can enable obese patients to return to daily life as soon as possible with a good clinical effect, and is expected to replace open surgery to become a new method of lumbar degenerative disease in young obese patients.

## Data Availability

The original contributions presented in the study are included in the article/Supplementary Material, further inquiries can be directed to the corresponding author.
